# Poor dietary diversity, wealth status and use of un-iodized salt are associated with goiter among school children: a cross-sectional study in Ethiopia

**DOI:** 10.1186/s12889-016-3914-z

**Published:** 2017-01-07

**Authors:** Zegeye Abebe, Ejigu Gebeye, Amare Tariku

**Affiliations:** 1 Department of Public Health Officer, Institute of Public Health, College of Medicine and Health Sciences, University of Gondar, Gondar, Ethiopia; 2 Department of Epidemiology and Biostatistics, Institute of Public Health, College of Medicine and Health Sciences, University of Gondar, Gondar, Ethiopia; 3 Department of Human Nutrition, Institute of Public Health, College of Medicine and Health Sciences, University of Gondar, P.O. Box: 196, Gondar, Ethiopia

**Keywords:** School children, Goiter, Salt iodine content, Ethiopia

## Abstract

**Background:**

Globally, more than two billion people are at risk of iodine deficiency disorders, 32% of which are school children. Iodine deficiency has been recognized as a severe public health concern in Ethiopia, however little is known about the problem. Therefore, this study aimed to assess the prevalence of goiter and associated factors among school children (6 to 12 years) in Dabat District, northwest Ethiopia.

**Methods:**

A school-based cross-sectional study was conducted from February 21 to March 31, 2016. A total of 735 school children were included in the study. A stratified multistage sampling followed by systematic sampling technique was employed to select the study participants. Thyroid physical examination was done and classified according to the World Health Organization recommendations as grade 0, grade 1, and grade 2. The level of salt iodine content was determined using the rapid field test kit. The value 0 parts per million (PPM), <15 PPM and ≥15 PPM with the corresponding color chart on the rapid test kit were used to classify the level of iodine in the sampled salt. A multivariable logistic regression analysis was employed to identify factors associated with goiter. Adjusted Odds Ratio (AOR) with a 95% Confidence Interval (CI) was calculated to show the strength of association. In multivariable analysis, variables with a *P*-value of <0.05 were considered statistically significant.

**Results:**

In this community, the overall prevalence of goiter was 29.1% [95% CI: 25.9, 32.6], in which about 22.4 and 6.7% had goiter grade 1 and grade 2, respectively. The age of children (AOR = 1.13; 95% CI: 1.01, 1.26), being housewife mother (AOR = 1.49; 95% CI: 1.08, 2.15), use of unprotected well water source for drinking (AOR = 6.25; 95% CI: 2.50, 15.66), medium household wealth status (AOR = 1.78; 95% CI: 1.18, 2.92), use of inadequately iodized salt (AOR = 2.79; 95% CI: 1.86, 4.19), poor dietary diversity score of the child (AOR = 1.92;95% CI: 1.06, 3.48) and medium maternal knowledge (AOR = 0.65; 95% CI: 0.42, 0.94) were significantly associated with goiter.

**Conclusions:**

The prevalence of goiter is higher in Dabat District, which confirmed a moderate public health problem. Therefore, regular monitoring of household salt iodine content, improving access to safe water, promoting the importance of diversified food for children is recommended to address the higher burden of iodine deficiency.

## Background

Iodine Deficiency (ID) is associated with a larger range of abnormalities which collectively named as ‘Iodine Deficiency Disorders (IDDs)’ reflecting thyroid dysfunction [1]. Particularly, goiter is used to describe an abnormal enlargement of thyroid gland mainly due to the adaptive response to low dietary iodine intake [2]. Due to their rapid growth and increased nutritional requirement, school children are considered as the most vulnerable segment of the community [3].

Globally, the total goiter rate is estimated to be 15.8% [4] and nearly two billion people are at risk of ID, while one-third lives in areas where natural sources of iodine is low [5]. Regarding the school children, about 32% are suffering from ID and related consequences [6]. Furthermore, the highest prevalence of ID is documented in Africa (42%) [1, 7]. Of the African countries, the largest burden is found in Ethiopia [8], according to which 39.9% of children are iodine deficient [9].

ID is found to severely impair the physical and mental development of children. The previous studies noted that iodine-deficient children perform poorly in school, suffered from the higher incidence of learning disabilities and lower intelligent quotient (IQ) [5, 10]. Besides to this, ID negatively affects working capacity, quality of life and economic productivity of the community at large [11]. Moreover, fatigue, poorer weight gain, cold intolerance, constipation, cretinism, congenital anomalies and iodine-induced hyperthyroidism is reported among iodine-deficient children [5, 12].

In addition to depletion of the iodine content of soil, the risk of developing ID is associated socio-demographic characteristics. Accordingly, age and sex of the child [13–15], larg famies [13], poor economic status [14], low maternal and paternal educational status [16–18], poor maternal knowledge about iodized salt [6, 16] and place of residence [19, 20] are significantly associated with ID. Furthermore, adding salt during food preparation [17], use of unpacked salt [21], storing salt for a longer duration, near to the fire, in open container, and exposing to heat and sunlight [14, 15] are found with increased odds of developing ID. Purchasing salt greater than 5 kg at once [14], consumption of food items containing goitrogens [15, 22, 23], and co-existing micronutrients deficiencies (iron, selenium and vitamin A deficiency) [24, 25] are also correlated with ID.

The government of Ethiopia has planned to achieve utilization of adequately iodized salt to at least 90% by the year 2015 [26]. Accordingly, the government designed National Nutrition Program, micronutrient guideline, and endorsed a proclamation for ensuring the availability of iodized salt. Moreover, Micronutrient Initiative (MI), Global Alliance for Improved Nutrition (GAIN), and United Nation Children’s Fund (UNICEF) are some of the international partners working with Federal Ministry of Health to rectify the child undernutrition [26, 27]. However, only 23.3% of the households used adequately iodized salt and ID continues as a critical public health problem in Ethiopia [9, 27].

Moreover, because of their higher vulnerability, measuring ID among school children is deemed to reflect the iodine status of the entire population [2]. However, little is known about IDDs in the northern part of Ethiopia, even the previous limited studies were done before and immediately after the implementation of universal salt iodization [28]. Therefore, this study aimed to assess the prevalence of goiter and associated factors among school children in Dabat District, northwest Ethiopia.

## Methods

### Study setting

A school-based cross-sectional study was conducted from February 21 to March 31, 2016, in Dabat District, northwest Ethiopia. The district is found 821 km from Addis Ababa, the capital city of Ethiopia. The district has 26 rural and four urban Kebeles (*smallest administrative unit in Ethiopia*). The altitude of the district ranges from 1000 to 2500 m above the sea level. The total population of 175,737 lives in the district. Cereals, such as maize, sorghum, wheat, and barley are the main staple crops cultivated in the district. The district has six health centers and 31 health posts. There are 82 schools in the district, 79 of which are primary schools. The Health and Demographic Surveillance System (HDSS) site was also located in Dabat District. The HDSS site has been running since 1996 and hosted by the University of Gondar. The surveillance site covers thirteen kebeles (four urban and nine rural kebeles) selected by considering different ecological zones (high land, middle land and lowland).

### Sample size and sampling procedure

All children aged 6–12 years who lived in HDSS site and attended primary school during the study period were eligible for the study. The sample size was calculated using Epi-info version 2.3 by using the following assumptions; the prevalence of goiter among school-aged children was 37.6% [28], 95% level of confidence and 5% margin of error. Finally, the sample size of 757 was obtained by considering 5% non-response rate and a design effect of 2. A multistage stratified sampling followed by systematic random sampling technique was employed to reach the study participants. Initially, schools were stratified into urban and rural. Of the total twenty-four primary schools in the HDSS site, five (one urban and four rural) schools with a total of 3429 students were selected using the lottery method. Number of students included in each school were proportionate-to-population size. Finally, a systematic sampling technique was employed to select the study subjects.

Physical examination was done for the selected child, after that using the child’s name, parent’s name and address, household visit was made by data collectors to gather the socio-demographic, the household utilization of iodized salt and dietary habit related characteristics of the child and the parents. Women who were majorly involved in food preparation of the household were selected as a respondent.

### Data collection instrument and procedure

A structured interviewer-administered questionnaire was used to collect data. The questionnaire was first prepared in English and was translated into the local language (Amharic) and back translated to English to maintain consistency by two BSc holder English teachers who are also native speakers of Amharic language. Pretest was done on five percent of the sample out of the study area. Two days training on techniques of interview, salt iodine content determination and thyroid physical examinationwas given for data collectors and supervisors. A total of nine data collectors (two health officers, an environmental health professional, and six permanent data collectors of the HDSS site) and three supervisors (two public health experts and a medical doctor) were involved in the study. Accordingly, the thyroid physical examination was undertaken by two Health Officers under the supervision of a medical doctor. Determination of salt iodine content was done by the trained environmental health professional. Daily supervision and feedback were carried out by the investigators and supervisors during the entire data collection period.

### Assessment of goiter and salt iodine content

The presence of goiter was assessed by the trained Health Officers with strict adherence to the standard procedures stipulated by the World Health Organization. Accordingly, goiter was defined as **grade 0** if no palpable mass in the neck was detected, **grade 1** if there was a mass in the neck consistent with palpable enlarged thyroid, but not visible when the neck was in the normal position, whereas **grade 2** was a swelling in the neck that was visible when the neck is in a normal position and is consistent with an enlarged thyroid when the neck is palpated (palpable and visible). Lastly, the child was deemed as having goiter when he/she had goiter of grade 1 or 2 [29].

A tablespoon of salt was collected from each household and the MBI international Rapid Test Kit (RTK) was used to determine the level of salt iodine content [16, 27, 29]. The small cup in the kit was filled with salt and made the cup surface flat. Two drops of test solution from white ampule were added to the surface of the salt by piercing the white ampoule with a pin and gently squeezing the ampule. The salt iodine content was determined within one minute by comparing the color developed on the salt with the color chart. The value 0 Parts per Million (PPM), <15 PPM and ≥15 PPM with the corresponding color chart on the rapid test kit were used to classify the level of iodine in the sampled salt. If no color appears, after 1 min, five drops of the recheck solution from red ampule was added to a fresh salt sample and followed by two drops of test solution on the same salt sample. Then, a comparison was done with the color chart indicators for salt iodine content [29].

### Assessment of dietary diversity

Determination of dietary diversity score (DDS) of the child was started by asking the mother to list all food consumed by the child in the previous 24 h preceding the survey. Then reported food items were classified into nine food groups, as starchy staples; dark green leafy vegetables; vitamin A rich fruits and vegetables; other fruits and vegetables; organ meat; flesh meat and fish; and egg [30]. Considering four food groups as the minimum acceptable dietary diversity, a child with a DDS of less than four was classified as having poor dietary diversity; otherwise, it was deemed to have good dietary diversity [30].

### Assessment of household wealth status and maternal knowledge

Household’s wealth index, adopted from EDHS 2011 [31], was determined using Principal Component Analysis (PCA) by considering the household assets, such as quantity of cereal products, type of house, livestock and agricultural land ownership. First, variables were coded between 0 and 1. Then variables entered and analyzed using PCA, and those variables having a communality value of greater than 0.5 were used to produce factor scores. Finally, the factor scores were summed and ranked into tertiles as poor, medium and rich.

Similarly, the knowledge of mothers towards iodized salt use was computed by using nine knowledge item questions, adopted by reviewing different literatures [7, 16, 28], including the health benefit of iodized salt, disorders resulted from ID, food sources of iodine, appropriate place for salt storage, time to add salt during food preparation, salt storage material and existence of law prohibiting selling of non-iodized salt in Ethiopia. Accordingly, the factor scores were summed and ranked into poor, medium and high.

### Data analysis

The collected data were checked and entered into Epi-info version 7 and exported to SPSS version 20 statistical software for analysis. Descriptive statics were carried out and the result was presented using text, tables and graph. A binary logistic regression model was fitted to identify factors associated with goiter. Variables with a *p*-value less than <0.2 in the bivariable analysis and those which frequently showed significant association with goiter in the previous studies were fitted into the multivariable logistic regression analysis and backward LR method was employed. Both Crude Odds Ratio (COR) and Adjusted Odds Ratio (AOR) with the corresponding 95% Confidence Interval (CI) were calculated to show the strength of association. In multivariable analysis, variables with a *p*-value of <0.05 were considered as statistically significant.

## Results

### Socio-demographic and economic characteristics

A total of 735 school children were included in the study, which makes a response rate of 97.1%. The median age of children was 10 years with Inter-quartile Range (IQR) of 3 years. About 54.1 and 61.6% children were females and lived in a family size of greater than five, respectively. Three-quarters of mothers were illiterate and 56.5% were outdoor workers. Most (80.7%) of the fathers were farmers (Table 1).

**Table 1:** Socio-demographic and economic characteristics of children and their parents, Dabat District, northwest Ethiopia 2016 (*n* = 735)

Variables	Frequency	Percentage
Sex of the child		
Male	337	45.9
Female	398	54.1
Residence		
Urban	155	21.1
Rural	580	78.9
Mother’s marital status		
Currently married	643	87.5
Currently unmarried^b^	92	12.5
Religion		
Orthodox	718	97.7
Muslim	2.3	2.3
Mother’s education		
Illiterate	561	76.3
Primary	100	13.6
Secondary and above	74	10.1
Father’s education		
Illiterate	405	55.1
Primary	238	32.4
Secondary and above	92	12.5
Mother’s occupation		
Housewife	320	43.5
Outdoor workers	415	56.5
Father’s Occupation		
Farmer	593	80.7
Merchant	42	5.7
Government employee	73	9.9
Others^a^	27	3.7
Family size		
< 6	282	38.4
≥ 6	453	61.6
Family history of goiter		
Yes	56	7.6
No	679	92.7
Source of drinking water		
Tap	168	22.9
Protected well	128	17.4
Unprotected well	34	4.6
Protected spring	271	36.9
Unprotected spring	134	18.2
Water treatment habit		
Yes	32	4.4
No	703	96.6
Wealth status		
Poor	253	34.4
Medium	237	32.2
Rich	245	33.3

^a^Daily laborer, student, pensioner

^b^Widowed, separated and single

### Utilization of iodized salt and consumption of iodine-rich food

A substantial proportion, (96.2%), of households used unpacked salt for food preparation, and about 49.8% households’ added salt at the beginning and middle of food preparation. Only one-third, (32.5%), of the households used adequately iodized salt (Table 2). Furthermore, about 85.2% of children had DDS of below four (mean ± SD of DDS of the children was 2.8 ± 0.67); while none of them were included fish in their diet within 7 days prior to the date of survey (Table 3).

**Table 2:** Household utilization of iodized salt and handling practices, Dabat District, northwest, Ethiopia, 2016 (*n* = 735)

Variables	Frequency	Percentage
Type of salt		
Packed	28	3.8
Unpacked	707	96.2
Addition of salt during food preparation		
At the beginning and the middle	366	49.8
At the end	369	50.2
Salt exposure to sunlight		
Yes	36	4.9
No	699	95.1
Washing of salt to remove impurities		
Yes	14	1.9
No	721	98.1
Quantity of salt purchased commonly		
Less than 1 kg	84	11.4
1 kg	456	62.0
2–5 kg	161	21.9
> 5 kg	34	4.6
Place of salt storage		
Near to the fire	72	9.8
Away from the fire	663	90.2
Salt storage material		
With closed container	696	94.7
Without closed container	39	5.3
Duration of household salt storage		
1–8 weeks	670	91.2
≥ 9 weeks	65	8.8
Salt iodine content		
0 ppm	21	2.9
1–14 ppm	475	64.6
≥ 15 ppm	239	32.5

**Table 3:** Consumption of iodine rich foods and goiterogenic substances among school-aged children, Dabat District, northwest, Ethiopia, 2016 (*n* = 735)

Variables	Frequency	Percentage
Milk and milk product		
Never	622	84.6
Once and more per week	113	15.4
Meat		
Never	600	81.6
Once and more per week	135	18.4
Egg		
Never	667	90.7
Once and more per week	68	9.3
Cabbage^b^		
Never	658	89.5
Once and more per week	77	10.5
Millet^b^		
Never	718	97.7
Once and more per week	17	2.3
Cereals commonly consumed^a^		
Maize	12	1.6
Teff	560	76.2
Sorghum^b^	509	69.3
Wheat	664	90.3
Millet^b^	9	1.2
Barely	517	70.3
DDS		
< 4 food groups	626	85.2
≥ 4 food groups	109	14.8

^a^Multiple responses

^b^Food items considered as containing goiterogenic substances

### Mother’s knowledge and attitude towards iodized salt use

One-third (33.2 and 29.1%, respectively) of the mothers had higher knowledge and favorable attitude towards iodized salt use. Regarding the cause of goiter, some of the mothers believed that it is due to contaminated water (34.5%), while 13.8 and 23.4% of them thought that it was because of genetic predisposition and drinking leftover water from a person who had a goiter, respectively. Only a quarter, (23.4%), of mothers considered that regular consumption of iodized salt can prevent goiter (Table 4).

**Table 4:** Mother’s knowledge and attitude towards iodized salt use, Dabat District northwest Ethiopia, 2016 (*n* = 735)

Variables	Frequency	Percentage
Knowledge		
Low	207	28.2
Medium	284	38.6
High	244	33.2
Attitude		
Low	267	36.3
Medium	254	34.6
High	214	29.1
Importance of iodized salt^a^		
Prevention of goiter	115	15.6
Growth and development	17	2.3
For health	380	51.7
I don’t know	327	44.5
The richest source of iodine^a^		
Egg	25	3.4
Meat	40	5.4
Milk and milk products	39	5.3
Iodized salt	68	9.3
Fish	8	1.1
Fruit and vegetables	9	1.2
I don’t know	611	83.1
Disorders of lack of iodine^a^		
Mental retardation	27	3.7
Goiter	182	24.8
Retarded growth	9	1.2
Abortion	9	1.2
Child mortality	2	0.3
I don’t know	519	70.6
All salts contain iodine		
Yes	77	10.5
No	220	29.9
I don’t know	438	59.6
Selling of non-iodized salt is inhibited in Ethiopia		
Yes	45	6.1
No	186	25.3
I don’t know	504	68.6
Test of iodized salt is different from unionized one		
Yes	185	25.2
No	276	37.6
I don’t know	274	37.3
Iodized salt has a harmful effect on health		
Yes	33	4.5
No	619	84.2
I don’t know	83	11.3
Sea salt contains iodine in the right quantities		
Yes	103	14
No	406	55.2
I don’t know	226	30.7
Ever seen people with swelling in the neck		
Yes	333	45.3
No	402	54.7
Causes of swelling in the neck (*n* = 333)^a^		
Genetics	46	13.8
Contaminated water	115	34.5
Drinking left over water	72	21.6
Sharing drinking material together	12	3.6
Drinking water contaminated by bird	6	1.8
Lack of iodine	34	10.2
I don’t know	47	14.1
Iodized salt prevents goiter (*n* = 333)		
Yes	78	23.4
No	160	48
I don’t know	95	28.6

^a^Multiple responses

### Prevalence of goiter among school children

**Fig. 1:**
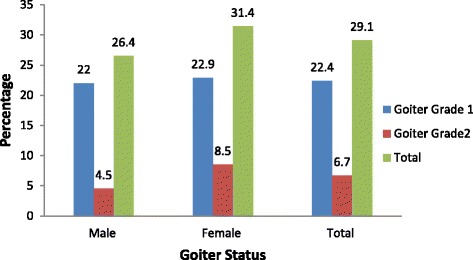
Prevalence and severity of goiter by sex of the children, Dabat District, northwest Ethiopia, 2016 (*n* = 735)

The overall prevalence of goiter was found to be 29.1% [95% CI: 25.9, 32.6]; one-fifth had grade-one goiter, while 6.7% had a grade two goiter. Moreover, goiter was more common among females (31.4%) than males (26.4%) (Fig. 1).

### Factors associated with goiter

Both bivariable and multivariable logistic regression analyses were done to see the effect of the selected characteristics on goiter. As it is presented in Table 5, child age, dietary diversity, residence, source of drinking water, mother’s and father’s education, father’s occupation, wealth status, the level of salt iodine content, and consumption of cabbage were the factors showed significant association with goiter in the bivariable analysis.

**Table 5:** Factors associated with goiter among school children, Dabat District, northwest Ethiopia, 2016 (*n* = 735)

Variables	Goiter status		Crude odds Ratio 95% C1	Adjusted odds Ratio 95% CI
	Yes #	No #		
Age	214	521	1.13 (1.02,1.26)	1.12 (1.01, 1.26)
Sex of the child				
Male	89	248	1.00	*
Female	125	273	1.28 (0.93, 1.76)	*
Residence				
Urban	29	126	1.00	*
Rural	185	395	2.04 (1.31, 3.16)	*
Mother’s education				
Illiterate	179	382	1.00	*
Primary education	19	81	0.50 (0.29, 0.85)	*
Secondary and above	16	58	0.59 (0.33, 1.05)	*
Father’s education				
Illiterate	127	278	1.00	*
Primary education	71	167	0.93 (0.66, 1.32)	*
Secondary and above	16	76	0.46 (0.26, 0.82)	*
Mother’s occupation				
House wife	102	218	1.27 (0.92, 1.74)	1.48 (1.02, 2.14)
Outdoor workers	112	303	1.00	1.00
Father’s occupation				
Farmer	191	402	1.00	*
Merchant	8	34	0.49 (0.26, 1.09)	*
Government employee	10	63	0.33 (0.17, 0.67)	*
Others	5	22	0.48 (.18, 1.28)	*
Family size				
< 6	73	209	1.00	*
≥ 6	141	312	1.29 (0.93, 1.80)	*
Family history				
Yes	17	39	1.07 (0.59, 1.93)	*
No	297	482	1.00	*
Source of drinking water				
Tap water	33	135	1.00	1.00
Protected well	35	93	1.54 (0.89, 2.65)	1.28 (0.66, 2.48)
Unprotected well	22	12	7.50 (3.37, 16.69)	6.38 (2.55, 16.01)
Protected spring	79	192	1.68 (1.06, 2.67)	1.14 (0.64, 2.03)
Unprotected spring	45	89	2.07 (1.23, 3.49)	1.41 (0.75, 2.65)
Household wealth index				
Poor	79	174	1.77 (1.18, 2.67)	1.27 (0.76, 2.12)
Medium	85	152	2.18 (1.45, 3.28)	1.75 (1.07, 2.87)
Rich	50	195	1.00	1.00
Type of salt				
Packed	7	21	1.00	*
Unpacked	207	500	1.24 (0.52, 2.97)	*
Addition of salt during food preparation				
At the beginning and the middle	95	271	0.74 (0.54, 1.01)	*
At the end	119	250	1.00	*
Salt exposure to sunlight				
Yes	10	26	0.93 (0.44, 1.97)	*
No	204	495	1.00	*
Salt storage				
Near to fire	20	52	0.93 (0.54, 1.60)	*
Away from fire	194	469	1.00	*
Salt storage				
With closed material	203	493	1.00	*
Without closed material	11	28	0.95 (0.47, 1.95)	*
Duration of salt storage				
1–2 months	192	473	1.00	*
> 2 months	22	43	1.27 (0.74, 2.19)	*
Salt iodine content				
0–14 ppm	175	321	2.80 (1.89, 4.13)	2.79 (1.86, 4.19)
≥ 15 ppm	39	200	1.00	1.00
Mother’s knowledge				
Poor	69	138	1.00	1.00
Medium	69	215	0.64 (0.43, 0.95)	0.65 (0.42, 0.94)
High	76	168	0.90 (0.61, 1.35)	0.99 (0.64, 1.55)
Mother’s attitude				
Poor	72	195	0.70 (0.47, 1.03)	*
Medium	68	186	0.69 (0.47, 1.03)	*
High	74	140	1.00	*
Cabbage consumption				
Never	200	460	1.00	*
Once and more per week	14	61	0.53 (0.29, 0.97)	*
DDS				
< 4 food groups	196	430	2.30 (1.35, 3.93)	1.92 (1.06, 3.48)
≥ 4 food groups	18	91	1.00	1.00

*Not appeared in the final model (not significant) using backward LR method

Nevertheless, the result of multivariable logistic analysis revealed that child age, dietary diversity, maternal occupation, knowledge on the use of iodized salt, household wealth status, the level of salt iodine content, and source of drinking water were significantly and independently associated with goiter. Consequently, with a year increase in age, the odds of having goiter were increased by 12% (AOR = 1.12; 95% CI: 1.01, 1.26). The likelihood of developing a goiter was 1.48 times (AOR = 1.48; 95% CI: 1.02, 2.14) higher among children whose mothers were housewives compared to children of mothers working outside the home.

In this study, the higher odds of developing a goiter were also observed among children living in the household using unprotected well water (AOR = 6.38; 95% CI: 2.55, 16.01) and with inadequately iodized salt (AOR = 2.77; 95% CI: 1.84, 4.15). As compared to the richer households, children from a household with medium wealth status were found at increased odds of having a goiter (AOR = 1.75; 95% CI: 1.07, 2.87). Likewise, the odds of developing a goiter among children with poor DDS were 1.92 times (AOR = 1.92; 95% CI: 1.06, 3.48) higher compared to their counterparts. However, the odds of having goiter were decreased by 35% (AOR = 0.65; 95% CI: 0.42, 0.94) among children whose mothers had medium knowledge towards iodized salt use as compared to children of mothers with poor knowledge (Table 5).

## Discussion

According to the WHO/UNICEF/ICCIDD established criteria, the area is classified as endemic for ID when it has a total goiter rate of more than 5% among school children (6–12 years). However, the public health importance of ID is defined as severe if the total goiter rate is greater than or equal to 30%; otherwise, it is deemed to have moderate and mild public health significance, if the magnitude ranged from 20.0 to 29.9%, and 5.0–19.9%, respectively [2].

Accordingly, the total goiter rate (29.1%) of this study area suggests a moderate public health significance of ID. But, compared to other local studies, this finding was lower than the national average (39.9%) [9] and what was reported from Lay-Armachiho District (37.6%) [28] and Goba District (50.6%) [13]. This is probably related to improvement in ensuring the availability of iodized salt throughout the country [27]. Currently, the government of Ethiopia gives priority to the implementation of mandatory salt iodization which is one of the proven strategies to address ID. For instance, China achieved a one-quarter (25.2%) reduction in total goiter rate following the implementation of universal salt iodization [32].

However, this prevalence was highest compared to reports of other developing countries, such as India (4.83–21.23%) [33, 34], Nigeria (13.2%) [35], and Saudi Arabia (11%) [19]. The discrepancy could be attributed to shorter duration of time in the implementation of universal salt iodization program in the study area compared to the latter study settings. In fact, thyroid size is slow to respond to change in iodine status [36]. In Ethiopia, universal salt iodization program has been implemented since 2011, though only one-third (32.5%) of the households utilize adequately iodized salt. As a result, the problem might still remain among children with larger thyroid size.

Similar to other reports elsewhere [13, 37], goiter was more prevalent among females in Dabat District. It is evident that females have a higher nutritional requirement for iodine, and reach to puberty earlier than males. In addition, it could be related to the effect of estrogen hormone on thyroid cell proliferation [38].

In this study, child age was independently associated with goiter. As the child’s age advances by a year, the probability of developing goiter was increased by 12%. The finding was supported by another study in Ethiopia [28] and Nigeria [35]. This is due to the fact that, iodine requirement increases with age. In addition, though dietary diversity is a proxy indicator of micronutrient adequacy of the diet [30], most of the children consumed undiversified diet in the study area.

The likelihood of developing goiter was 1.48 times higher among children whose mothers were housewives compared to children of the mothers working outside home. More than three quarters, (77.5%), of the housewives in this study were illiterate. Illiterate mothers might have lesser capacity to understand the adverse consequences of ID and the food sources of iodine to appropriately feed their child. The previous reports also affirmed that undiversified diet and other poor feeding practices were commonly observed among children of illiterate mothers [31, 39].

In line with this fact, this study also showed increased odds of developing a goiter among children with poor DDS compared to their counterparts. In the case of the communities with cereal based monotonous dietary habit, most of the children suffered from ID and other co-existed micronutrient deficiencies, like vitamin A and iron deficiency [24, 40–42].

Household’s source of drinking water was significantly associated with goiter. Accordingly, the higher odds of having goiter were noted among children from households using the unprotected source of water. The finding was in agreement with the previous studies of other developing countries [10, 43–45], in which contamination of drinking water with Coliforms and E. Coli contributes to the development of goiter. The current study revealed that the majority, (96.6%), of the households did not treat water to make it safer for consumption.

In this study, household wealth status was inversely associated with risk of developing a goiter. The odds of developing goiter among children from households with medium wealth status were higher compared to children from richer households. The finding was in line with the studies done elsewhere [14, 40, 46]. Obviously, wealth status determines the household’s food purchasing power and food security status [47]. Accordingly, rich households can access a variety of food which ultimately improves the child’s dietary diversity. In addition, utilization of un-iodized salt is common among poor households [44].

It was documented that, poor maternal knowledge towards iodized salt use was positively associated with goiter [32, 40, 45]. Similarly, the odds of having goiter were reduced by 35% among children whose mothers had medium knowledge compared to those children whose mothers had poor knowledge. Boosting mother’s knowledge of iodized salt use is an important step to ensure appropriate utilization of iodized salt at the household level [48].

Finally, inadequate salt iodine content of the household was associated with the higher odds of developing a goiter. This finding was in line with another report from Ethiopia [28] and Saudi Arabia [19]. Implementation of universal salt iodization is the most cost effective and proven intervention to eliminate IDDs [49, 50], in spite of this fact only one-third of the households utilized adequately iodized salt and majority of children were found with poor dietary intake of iodine rich food.

The study was conducted using relatively large sample size and in a well-defined population representing the northwest part of Ethiopia. In addition, the study also determined the recent iodized salt consumption. However, some of the limitations of this study should be taken into consideration. First, the study did not include biochemical markers of recent iodine status. Second, eventhough adequate training was given to field assistants (data collectors and supervisors) and mothers were clearly informed about the objectives of the study, still, there might be social desirability bias in responding type of salt use and handling practice.

## Conclusion

The prevalence of goiter was higher in the study area which confirmed a moderate public health problem. Child age, dietary diversity score, maternal occupation, knowledge, the household source of drinking water, wealth status, and level of salt iodine content were significantly associated with goiter. Hence, regular monitoring of household salt iodine content, improving access to safe water and promoting dietary diversification is recommended to address the higher burden of ID. Finally, conducting further studies by including biochemical markers and determining salt iodine level using iodometric titration is recommended for the researchers.

## Abbreviations

DDS: Dietary diversity score
EDHS: Ethiopia Demography and Health Survey
FAO: Food and Agricultural Organization
HDSS: Health and Demography Surveillance System
ICCIDD: International Committee of Control of Iodine Deficiency Disorder
ID: Iodine Deficiency
IDD: Iodine Deficiency Disorder
PCA: Principal Component Analysis
PPM: Parts per Million
TGR: Total goiter rate
UNICEF: United Nation Children’s Fund
WHO: World Health Organization
